# The first report of molecular characterized BRD4-NUT carcinoma in Brazil: a case report

**DOI:** 10.1186/s13256-019-2213-6

**Published:** 2019-09-07

**Authors:** Leandro J. C. Oliveira, Aline B. L. Gongora, Marcela T. Latancia, Felipe G. Barbosa, João Vitor A. M. Gregorio, Leonardo A. Testagrossa, Mariane T. Amano, Olavo Feher

**Affiliations:** 1 Centro de Oncologia, Hospital Sírio Libanês, Rua Dona Adma Jafet, 91. 2nd floor. Building A, São Paulo, 01308-050 Brazil; 2 Instituto de Ensino e Pesquisa, Hospital Sírio Libanês, São Paulo, Brazil; 3 Serviço de Medicina Nuclear, Hospital Sírio Libanês, São Paulo, Brazil; 4 Serviço de Oncologia, Instituto do Câncer do Estado de São Paulo, Universidade de São Paulo, São Paulo, Brazil; 5 Serviço de Anatomia Patológica, Hospital Sírio Libanês, São Paulo, Brazil

**Keywords:** NUT midline carcinoma, Poorly differentiated squamous cell carcinoma, Molecular pathology, Targeted therapy

## Abstract

**Background:**

NUT midline carcinoma is a rare and aggressive subset of squamous cell carcinoma, which is characterized by the translocation of nuclear protein in testis gene that is mostly fused with bromodomain and extraterminal family proteins. We describe here the first Brazilian case of NUT midline carcinoma with BRD4-NUT fusion detected in a next-generation sequencing panel and we present the clinical evolution of this patient.

**Case presentation:**

A 42-year-old Caucasian man was diagnosed with poorly differentiated squamous cell carcinoma of the left maxillary sinus, with negative *in situ* hybridization for Epstein–Barr encoding region and human papillomavirus genotyping. He received induction therapy, chemoradiotherapy with weekly systemic chemotherapy, and, concurrently, weekly intra-arterial chemotherapy. New imaging evaluation, 1 month after the end of the last treatment, revealed a good partial response in the primary lesion. However, positron emission tomography-computed tomography showed multiple suspicious lesions in his bones and lungs, which were histologically confirmed. He died exactly 2 months after metastatic disease was diagnosed.

**Conclusions:**

NUT midline carcinoma is usually very aggressive. Currently, there is no standard of care for treatment of NUT midline carcinoma. The definitive diagnosis must be by demonstration of *NUTM1* rearrangement. Immunohistochemical staining of greater than 50% of tumor nuclei on formalin-fixed paraffin-embedded tissue using the monoclonal rabbit antibody to NUT (clone C52B1), has a specificity of 100%, and sensitivity of 87% for the diagnosis of NUT midline carcinoma. Our case is the first Brazilian case of NUT midline carcinoma with BRD4-NUT fusion.

## Background

NUT midline carcinoma (NMC) is a rare and aggressive subset of squamous cell carcinoma, which is characterized by the translocation of nuclear protein in testis (*NUT*) gene that is mostly fused with bromodomain and extraterminal (BET) family proteins, such as BRD3 and BRD4 [1], generating BRD3-NUT and BRD4-NUT, respectively. A non-BET member, nuclear receptor binding SET domain 3 (NSD3), was also seen to be fused with *NUT* (NSD3-NUT) [2, 3], but this seems to be less frequent than BRD3-NUT and BRD4-NUT. The majority of the cases occur in the head, neck, and mediastinum, although there are reports involving the bladder, pancreas, adrenal gland, kidney, and salivary gland [4]. NMC differs from other carcinomas, which usually have complex karyotypes, because it is characterized by a few or a single translocation, most often the BRD4-NUT: t(15;19)(q14;p13.1) [4].

Until 2011, NMC was considered an extremely rare carcinoma, with only 28 reported cases worldwide [5]. Since 2012, the number of diagnosed cases increased enormously; this was identified in a review in the International NUT Midline Carcinoma Registry, which detected 48 cases of NMC from 2011 to 2014, of which 86% were BRD4-NUT positive [6]. This review suggests that NMC is probably underdiagnosed.

We describe here the first Brazilian case of NMC with BRD4-NUT fusion detected in next-generation sequencing (NGS) panel and we present the clinical evolution of this patient. Until recently, to the best of our knowledge, no cases of NMC had been reported in Latin America [4, 7]. Salles *et al.* described four patients with typical NMC histopathology and NUT positivity in the nucleus [8]. However, the fusion was not molecularly described. Since there is a clear failure in detecting NMC in patients, and further studies are required to provide better chances for these patients, we understand that there is a need to report and better characterize cases of NMC.

## Case presentation

**Fig. 1:**
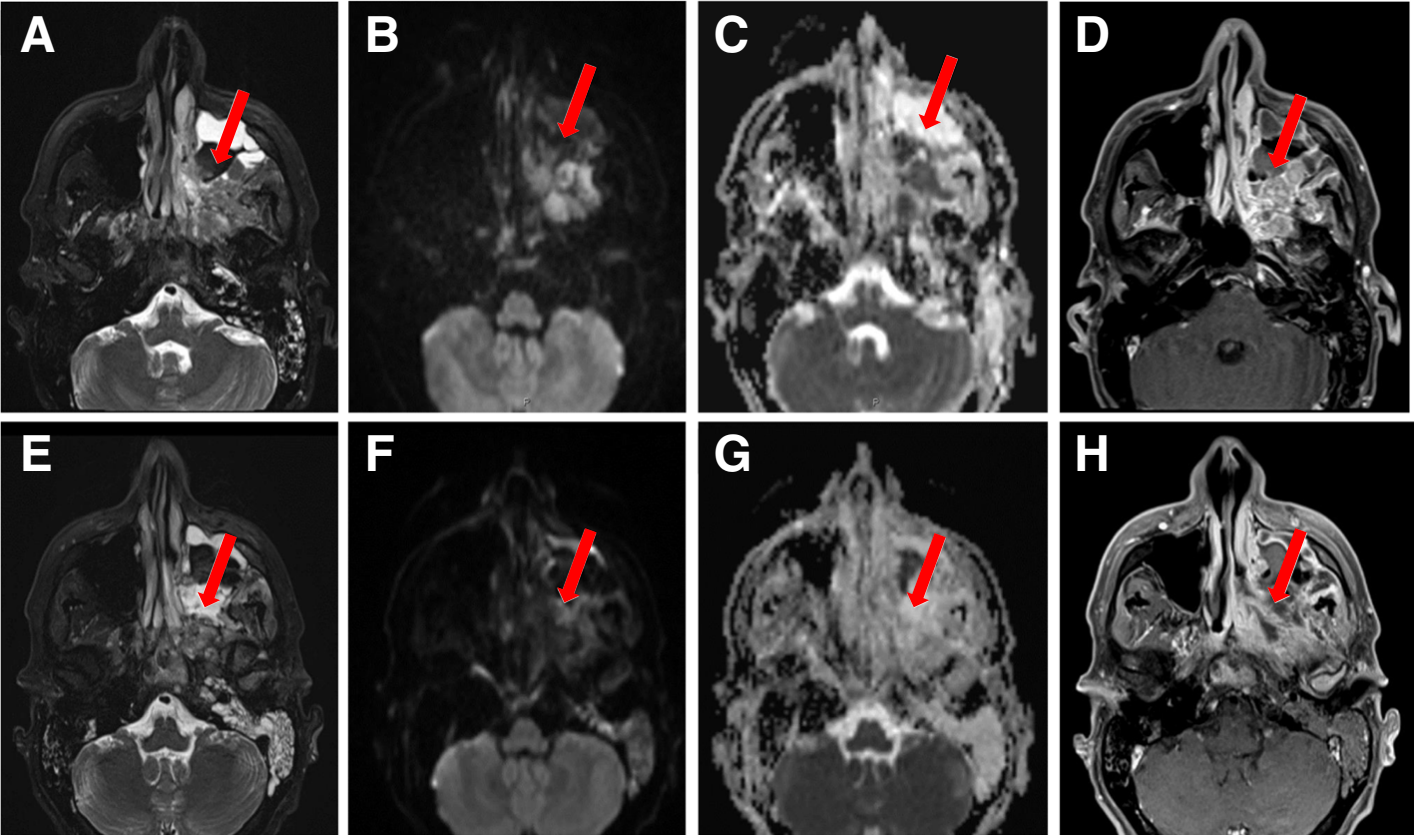
Local tumor response seen in head and neck magnetic resonance images. Magnetic resonance images from baseline before second-line chemotherapy (**a**–**d**) showing locally advanced left maxillary sinus mass (*arrows*), infiltrating adjacent sinuses and skull base foramina. Follow-up magnetic resonance images (**e**–**h**) showing significant tumor shrinkage (*arrows*) with areas of necrosis, representing local morphological partial response

A 42-year-old Caucasian man presented in December 2016 with productive cough, facial pain, and rhinorrhea. He is an engineer, who does not smoke tobacco, and he had no significant premorbid conditions. There was no history of prescription drug use, and no significant family history. Neurological, cardiovascular, respiratory, and abdominal examinations were normal, except for tenderness of the face elicited by palpation. He was first diagnosed as having acute rhinosinusitis and treated with antibiotics (details were not available), without improvement. Due to symptoms persistence, magnetic resonance imaging (MRI) of his face was ordered. This study revealed an expansive irregular heterogeneous lesion (4.5 × 3.5 × 4.0 cm), with its central portion located on the interface between the left maxillary sinus and the pterygopalatine fossa. This lesion invaded medially the nasal cavity and posterosuperiorly the left orbit apex with an intracranial extension through the inferior orbital fissure (Fig. 1a–d). There was no lymphadenopathy and no perineural invasion. A biopsy demonstrated poorly differentiated squamous cell carcinoma with negative *in situ* hybridization for Epstein–Barr encoding region (EBER). Human papillomavirus (HPV) genotyping test was negative as well. Positron emission tomography-computed tomography (PET-CT) was negative for nodal and systemic metastases. His total leukocyte and platelet counts, as well as hemoglobin levels, were all within normal limits. His biochemical parameters, including serum electrolytes, renal function test, and liver function test, were also normal.

**Fig. 2:**
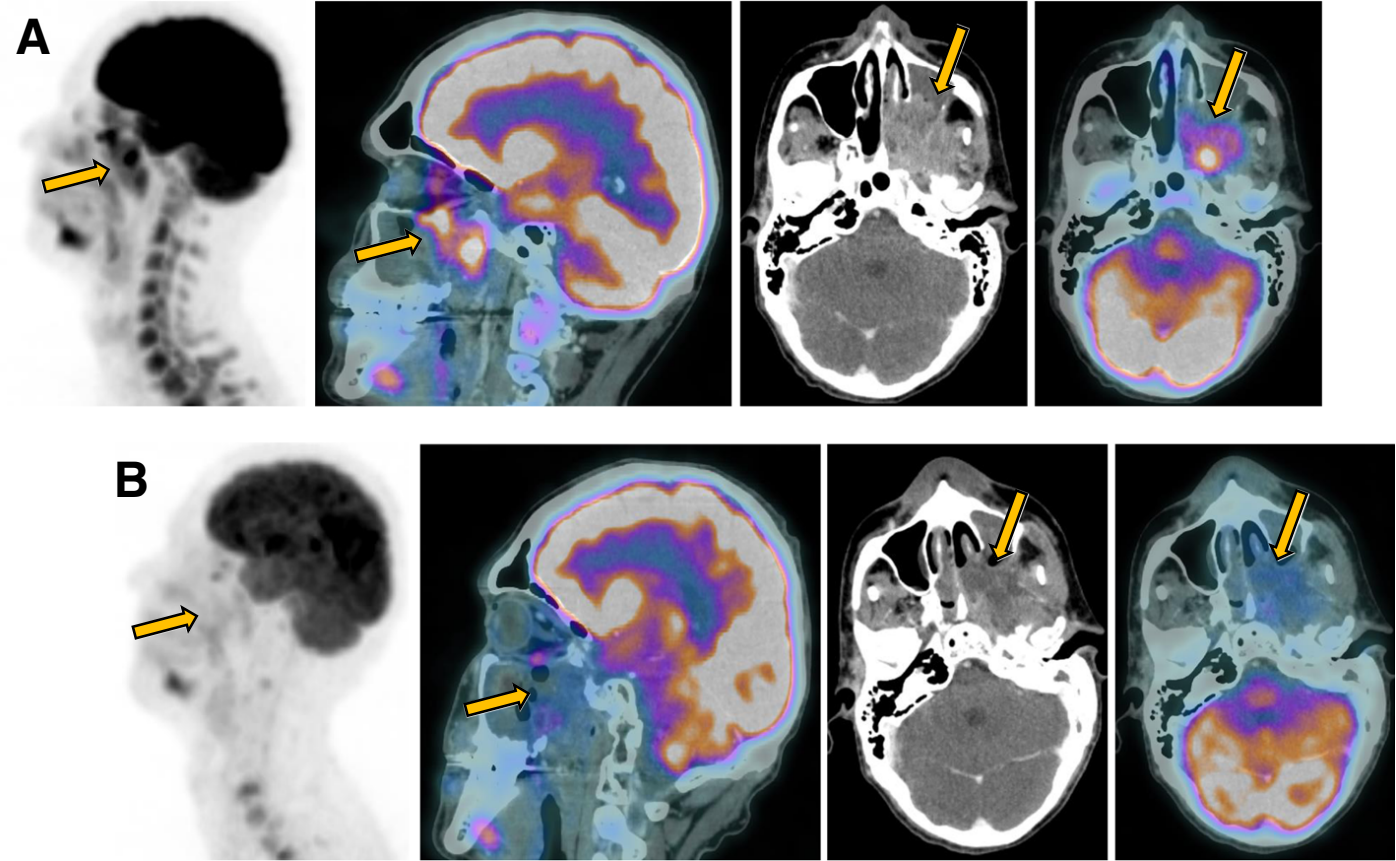
Local complete metabolic response seen in ^18^F-fluorodeoxyglucose positron emission tomography-computed tomography. Fluorodeoxyglucose-positron emission tomography-computed tomography head and neck images from baseline before second-line chemotherapy (**a**) showing locally advanced left maxillary sinus enhanced mass (*arrows*) with fluorodeoxyglucose avidity. Positron emission tomography-computed tomography of 1 month after end of treatment (**b**) confirming morphological shrinkage of the left maxillary sinus with extensive necrosis (*arrows*) without any fluorodeoxyglucose uptake, representing local complete metabolic response

He received, from January 2017 to April 2017, induction therapy with docetaxel 75 mg/m^2^ at day 1, cisplatin 100 mg/m^2^ at day 1, and fluorouracil (5-FU) 1000 mg/m^2^ per day at day 1 to day 4 (DCF) for six cycles every 3 weeks, with clinical benefit and stable disease by MRI and PET-CT (Figs. 1 and 2). Chemoradiotherapy (radiotherapy 35 fractions – 70 Gy) with weekly systemic chemotherapy based on carboplatin 1.5 area under the curve (AUC), paclitaxel 45 mg/m^2^, and cetuximab 400 mg/m^2^ was administered for 7 weeks from May 2017 to July 2017. Concurrently, weekly intra-arterial chemotherapy with cisplatin 150 mg/m^2^ was performed for 5 weeks with grade 2 myelotoxicity and nausea.

**Fig. 3:**
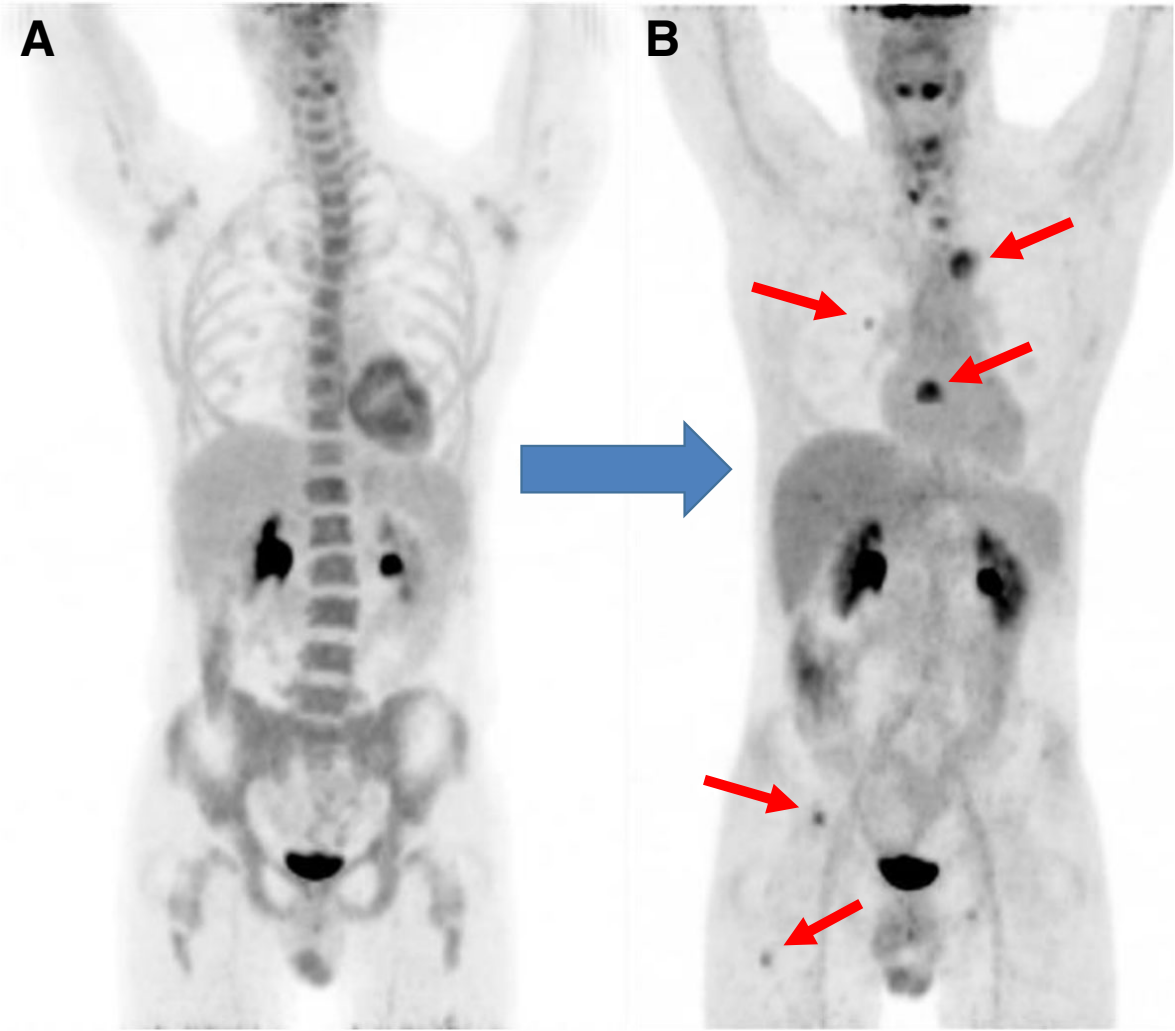
Systemic disease progression evidenced in ^18^F-fluorodeoxyglucose positron emission tomography-computed tomography maximum intensity projection images. Maximum intensity projection fluorodeoxyglucose-positron emission tomography-computed tomography whole-body images from baseline before second-line chemotherapy (**a**) and 1 month after end of treatment (**b**). Image a shows no evidence of systemic disease. Image b shows disease progression with new bone and pulmonary fluorodeoxyglucose-avid metastasis (*arrows*)

**Fig. 4:**
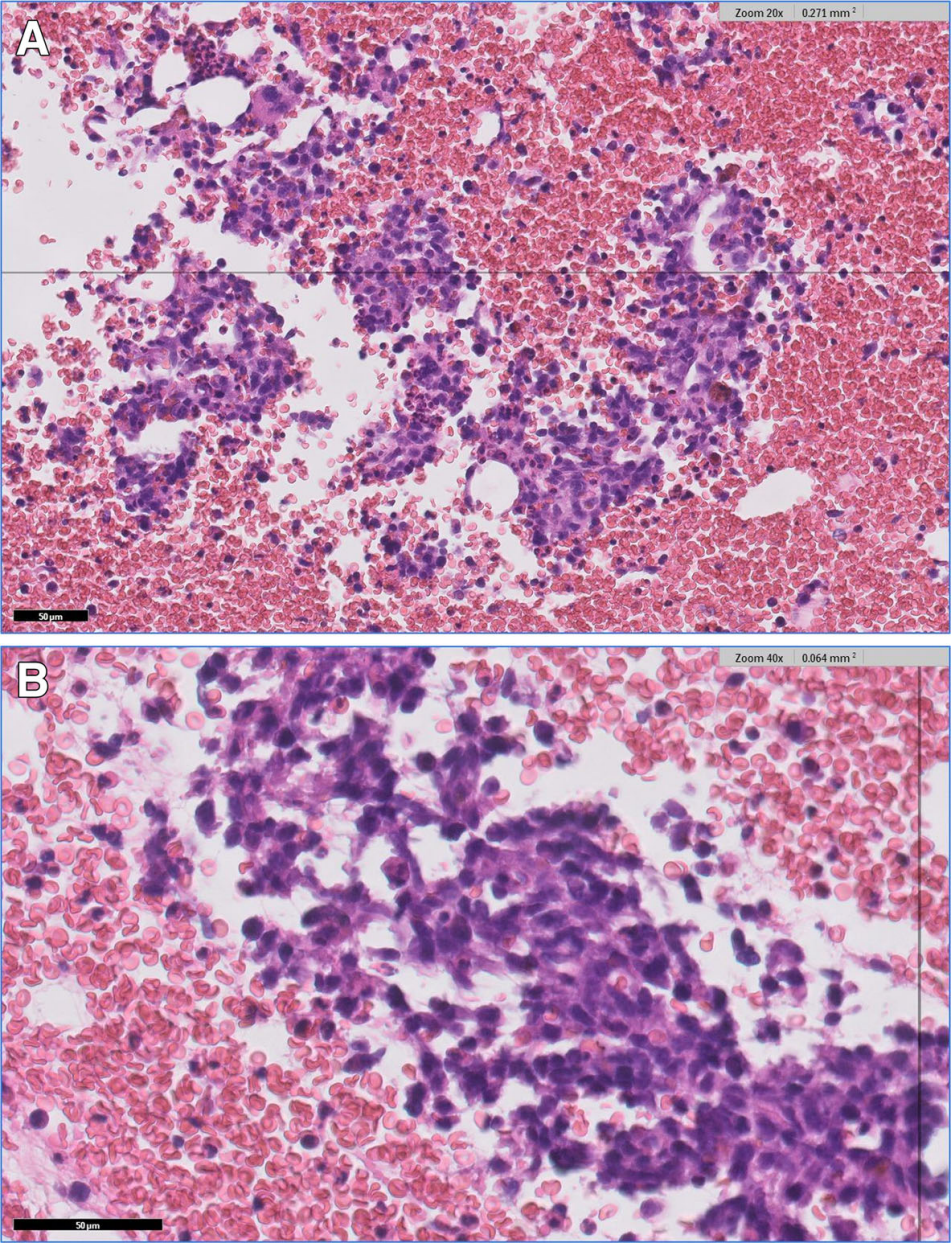
**a** Bone metastasis of poorly differentiated carcinoma showing neoplastic epithelioid clusters with small cells (hematoxylin and eosin, × 200). **b** Higher magnification of the neoplastic clusters (hematoxylin and eosin, × 400)

**Fig. 5:**
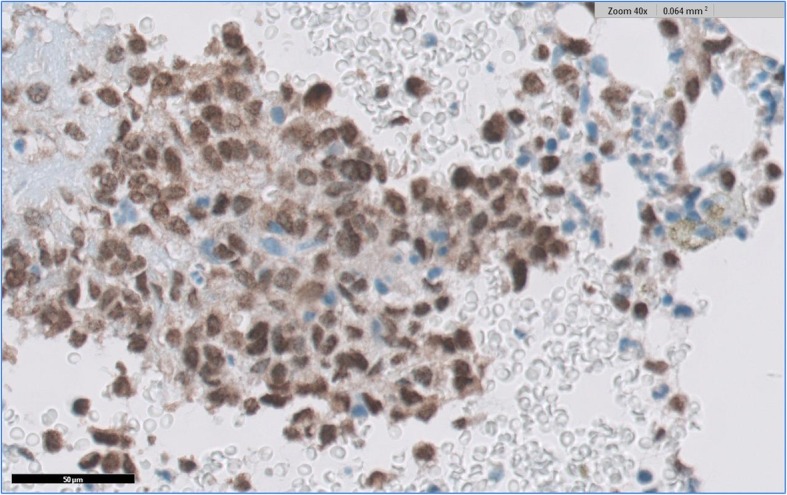
NUT immunohistochemistry staining positively in neoplastic nuclei

New imaging evaluation, 1 month after the end of the last treatment, revealed a good partial response in the primary lesion (Figs. 1e–h and 2). However, PET-CT showed multiple suspicious lesions in his bones and lungs (Fig. 3), which were histologically confirmed (Fig. 4a, b). Programmed death-ligand 1 (PD-L1) expression by immunohistochemistry (IHC) was negative (SP263 Ventana). In order to look for other therapeutic possibilities, a formalin-fixed paraffin-embedded (FFPE) tumor biopsy was sent to the Foundation Medicine with patient consent. Deoxyribonucleic acid (DNA) extracted from FFPE and hybrid capture-based NGS was applied to perform the FoundationOne™ test, which comprises a panel of 315 genes known to carry somatic mutations in human solid tumors, as well as introns of 28 genes involved in rearrangements. The test showed a rearrangement of BRD4-NUT characterizing NMC. No other mutations were found; microsatellite stability and low mutational burden were reported. The IHC for NUT protein was performed and stained positively in neoplastic nuclei (Fig. 5).

Due to the lack of available clinical trials in the country for this highly aggressive tumor, our patient received two more cycles of chemotherapy, but, unfortunately, without any response. He died exactly 2 months after metastatic disease was diagnosed.

## Discussion

We describe the first report of a molecular characterized BRD4-NUT carcinoma in Brazil and Latin America. Our patient showed a significant partial response in the primary lesion but progressed with multiple metastatic lesions in bone and lungs.

Reports of NMC have been increasing since it was originally described as a genetically defined entity in 2004 [9]. It can affect individuals of all ages, despite the initial thought that it only affected children and young adults. The median age of diagnosis is 24 years. The most common primary sites are thorax, in 50% of the patients, and head and neck, in 39% of patients. There are other primary sites (non-midline, non-thoracic, or head and neck) described in reports of NMCs, such as soft tissue or bone, which can account for 11% of all cases [10]. In Brazil, lung and head and neck cancers are among the ten most frequent cancers, especially in men [11]. However, until now, there were no cases of NUT-BRD4 NMC confirmed in Brazil, probably due to underdiagnosis.

NMC is usually very aggressive. Not rarely, it is metastatic at diagnosis and has poor survival rates. An analysis of a clinical database with 63 patients with NMC and outcome data of 54 patients showed a median overall survival (OS) of 6.7 months. The 2-year OS was 19% [12]. A systematic review and individual patient data analysis of 119 cases showed median OS of only 5 months. Radiotherapy and chemotherapy had a significant impact on OS, while surgery, BET inhibitors, and histone deacetylase inhibitors (HDACi) did not affect survival significantly [13].

As previously discussed, the karyotypes of NMC are not complex; they frequently have a clonal translocation of the *NUTM1* gene, classically t(15;19)(q14;p13.1), which places NUT in the frame with BRD4. This feature differs from other carcinomas because the latter develop over years due to an accumulation of mutations and genetic aberrations [14]. The BRD4-NUT fusion oncoproteins drive a series of disruptions of gene expression through chromatin remodeling [15], overexpression of MYC [16], and sequestration of the activated form of p53 [17]. Another possibility is the fusion of NUT with BRD3 or NSD3, which are functionally related to BRD4. Therefore, recruitment of NUT to the chromatin through the BET family proteins is probably necessary for the oncogenesis of NMC [17].

On histological examination, NMC can have a broad morphologic range, in many cases resembling squamous cell carcinoma, in others showing small undifferentiated cells with or without areas of abrupt keratinization. Therefore, diagnosis should not be assumed just based on histological aspect [18].

The definitive diagnosis must be by demonstration of the *NUTM1* rearrangement. There is no consensus on which pathology material of the patient should be submitted to molecular tests. Some authors suggested performing IHC or molecular tests in all poorly differentiated non-cutaneous carcinomas, with or without squamous differentiation, with a monomorphic appearance. Glandular tumors do not need to be tested. Viral etiology, such as HPV or Epstein–Barr virus (EBV), is not associated with NMC and can be used to rule this diagnosis out. However, NMC can be strongly positive for p16, despite its negativity for HPV; therefore, it should not be used for excluding NMC [14].

IHC staining of greater than 50% of tumor nuclei on FFPE tissue using the monoclonal rabbit antibody to NUT (clone C52B1) has a specificity of 100%, and sensitivity of 87% for the diagnosis of NMC. Some germ cells tumors can also stain, but usually focally (< 10%) [19]. Confirmatory molecular tests are not mandatory. However, they can be used if the C52B1 antibody is not available or to identify the fusion partner to NUT, which may have clinical and prognostic relevance. For example, NSD3 or BRD3-NUT-positive NMC was associated with a better OS than those with BRD4-NUT, especially in non-thoracic NMC [14]. The assays that can be used for this purpose are fluorescence *in situ* hybridization (FISH), reverse transcriptase-polymerase chain reaction (RT-PCR), cytogenetics, and NGS-based approaches. Molecular diagnostic methods have limitations, such as the cost and access to quality services [14].

Currently, there is no standard of care for treatment of NMC. Incomplete or no surgical resection and no initial radiotherapy have been associated with shorter progression-free survival and OS. However, no chemotherapeutic regimen has been associated with better outcomes [12].

Targeted therapies are in development encompassing different strategies (Table 1). A first-in-class BET inhibitor, birabresib, a selective inhibitor of BRD2, BRD3, and BRD4 was evaluated in a recently published phase Ib trial. In this trial with 43 patients, 10 of them with NMC, partial response was observed in 3 out of these 10 patients with NMC.

**Table 1:** NUT midline carcinoma clinical trials available at Clinicaltrials.gov

Trial	Type of study/ population	Regimen	Mechanism of action	Status
NCT01587703	Phase I/II – Diagnosis of NMC determined by IHC and/or detection of NUT gene translocation by FISH, treatment naïve or with prior therapy	GSK525762	BET protein inhibitor	Active, not recruiting
NCT03702036	Compassionate use – Diagnosis of NMC determined by IHC and/or detection of NUT gene translocation by FISH, with no other satisfactory alternative treatment	GSK525762 (molibresib)	Inhibitor of the binding of BET proteins to acetylated histones	Available
NCT02307240	Phase I – Diagnosis of an advanced solid tumor such as breast cancer **or NMC**, that has progressed despite standard therapy, or for which no standard therapy exists	CUDC-907	HDAC and PI3K Inhibitor	Active, not recruiting
NCT02698176	Phase IB – Diagnosis of one of the following advanced solid tumors for which standard therapy either does not exist or has proven ineffective, intolerable, or inacceptable for the participant: **NMC**;TNBC; NSCLC; or CRPC	MK-8628	BET protein inhibitor	Terminated due to limited efficacy
NCT02516553	Phase I – Diagnosis of advanced unresectable and/or metastatic solid tumor, refractory to conventional treatment or for whom no therapy of proven efficacy exists, or who are not amenable to standard therapies. Part Ib includes patients with SCLC, CRPC, CRC or **NMC**	BI 894999	BET protein inhibitor	Recruiting
NCT02259114	Phase I – Advanced or metastatic: TNBC, NSCLC, CRPC, pancreatic ductal adenocarcinoma, **NMC,** for which standard therapy either does not exist or has proven ineffective, intolerable or inacceptable for the patient	OTX015/MK-8628 (birabresib)	BET protein inhibitor	3/10 patients with NMC with PR with duration of 1.4 to 8.4 months
NCT02711137	Phase 1/2 – Histologically or cytologically confirmed diagnosis of relapsed or refractory advanced or metastatic malignancies (solid or hematologic)	INCB057643	BET protein inhibitor	Terminated due to safety issues
NCT02431260	Phase 1/2 – Any advanced solid tumor or lymphoma; acute leukemia, myelodysplastic syndrome, myelodysplastic /myeloproliferative neoplasms, myelofibrosis, and multiple myeloma	INCB054329	BET protein inhibitor	Terminated by the sponsor due to pharmacokinetics variability [20]
NCT02369029	Phase 1 – Patients with advanced tumors refractory to any standard treatment, with no standard therapy available or in whom standard therapy is not a therapeutic option	BAY1238097	BET protein inhibitor	Terminated because of dose-limiting toxicities at a dose below targeted drug exposure [21]

*BET* bromodomain and extraterminal, *CRPC* castration-resistant prostate cancer, *CRC* colorectal cancer, *FISH* fluorescence *in situ* hybridization, *HDAC* histone deacetylase, *IHC* immunohistochemistry, *NMC* NUT midline carcinoma, *NSCLC* non-small cell lung cancer, *PI3K* phosphoinositide 3-kinase, *PR* partial response, *SCLC* small cell lung cancer, *TNBC* triple negative breast cancer

In addition, other targets have been studied. A dual histone deacetylases (HDAC) and phosphoinositide 3-kinases (PI3K) inhibitor, CUDC-907, was tested in a preclinical trial and showed some positive results. The rational was that inhibition of HDAC and PI3K, upstream regulators of MYC, could reduce MYC protein levels, a key oncogenic target in NMC, and ultimately lead to growth suppression and cell death [22]. Activity of these drugs in NMC was shown *in vivo* and *in vitro*, associated with decreased MYC protein levels. This activity should be further studied in clinical studies.

This is the first Brazilian case of NMC with BRD4-NUT fusion. Because of this diagnosis, the routine in our Pathology department has changed and the IHC for NUT protein is now validated and performed in all cases of poorly differentiated carcinomas, with or without keratinization, especially those occurring in young patients, never smokers, with unusual IHC patterns, atypical morphology and/or occurring in midline sites.

## Conclusion

Our case is the first Brazilian case of NMC with BRD4-NUT fusion detected in a NGS panel. The diagnosis of NMC holds importance for prognostic value as well as for clinical trials with targeted therapy. This report reinforces the need for definitive diagnosis of this subtype of low differentiated carcinoma, and highlights the IHC evaluation, the most available and accessible method, as an important diagnostic tool.

## Data Availability

Not applicable.
